# Compliance With Epidemic Prevention Guidelines Among Wuhan Citizens Under the Stressors of the COVID-19 Pandemic: A Cross-Sectional Multidistrict Comparative Analysis

**DOI:** 10.3389/fpsyg.2022.808617

**Published:** 2022-03-23

**Authors:** Xuelian Wang, Yuwei Cai

**Affiliations:** ^1^School of Journalism and Communication, Wuhan Sports University, Wuhan, China; ^2^Graduate School, Wuhan Sports University, Wuhan, China

**Keywords:** COVID-19, compliance with epidemic prevention guidelines, multidistrict, anxiety, conscientiousness

## Abstract

The current study aims to compare compliance with the COVID-19 prevention guidelines among citizens from 13 districts in Wuhan and to explore the influence of individual-level psychological factors and district-level factors on compliance. A total of 811 participants (52% females) from 13 districts in Wuhan were invited to complete surveys regarding demographics, psychosocial factors and compliance with epidemic prevention guidelines. Individual-level characteristics were combined with district-level measures to create multilevel predictive models of compliance with prevention guidelines, and used the Hierarchical Linear Model (HLM) to analyze the data. Findings revealed that there were significant differences in the compliance of citizens from 13 districts of Wuhan (*F* = 5.65, *P* < 0.001). Hierarchical linear model analysis revealed that the risk factors case growth rate, COVID-19-related perceived stress, anxiety, significantly negatively predicted compliance. Hope and conscientiousness significantly positively predicted compliance with prevention guidelines, and the negative predictive effect of anxiety disappeared. Overall, we found significant differences in compliance with prevention guidelines among different districts. Risk factors at the individual level have had a negative impact on individuals’ compliance with prevention guidelines, but this impact can be mitigated by the positive role of personal protective factors such as conscientiousness and hope.

## Introduction

The 2019 coronavirus disease (COVID-19) epidemic is a global health threat. The outbreak was first revealed in late December 2019 in the city of Wuhan in Hubei Province. Since then, the number of cases has continued to escalate exponentially within and beyond Wuhan, spreading to all 34 regions of China by 30 January 2020 (Wang et al., 2020). Since the outbreak, response efforts by the Chinese government have been swift. To prevent further spread, protect the health of the public, and maintain the normal order of production, life and traffic, the Chinese Center for Disease Control and Prevention, under the guidance of the National Administration of Disease Prevention and Control, jointly launched the “Guidelines for Public Protection of Pneumonia Caused by the 2019 Coronavirus” with People’s Medical Publishing House. The purpose is to publicize knowledge about public protection from COVID-19 that is correct, authoritative and professional to prevent the public from panic and to ensure correct understanding, good protection and health maintenance. However, during the epidemic lockdown, the public’s compliance with the guidelines was inevitably affected by many factors. Existing studies have shown that negative factors such as how individuals perceived the difficulty and severity of disease, exposure factors, protective resources negatively affect behavior compliance (Zhou and Luo, 2009). Previous research has revealed a profound and wide range of psychosocial impacts on people at the individual, community, and international levels during outbreaks of infection (Wang et al., 2020).

As the severity of the epidemic is not consistent in different districts of Wuhan, there may be some differences in the protective behaviors of citizens in different districts. The people of Wuhan, Hubei province, China—where the COVID-19 outbreak was first noted, and which has had the highest number of confirmed cases and deaths in China—experienced collective bereavement and grief (Cao et al., 2020) when their city was forced into complete lockdown for 76 days. In addition, changing health alerts and overwhelming media coverage of the spread of COVID-19 in the city have increased fear, anxiety and even stigma among urban residents, all of which can have profound effects on mental health (Li et al., 2021). In this environment, Wuhan citizens’ protective behavior is affected by many factors. In the early stage of the outbreak, there was a great difference in the severity of the epidemic among the districts of Wuhan, as well as in the medical resources and the number of confirmed cases, so it was necessary to conduct hierarchical research.

## Theoretical Frameworks and Aims

### The Impact of Risk Factors on Individuals’ Compliance With Epidemic Prevention Guidelines

Based on resource conservation theory, resource loss can lead to adverse reactions for individuals. When people face despair from resource depletion, it may also trigger the individual defense mechanism with the aim of self-protection that results in showing some irrational behavior, but people will use critical resources in response to the current environment stress scenarios to alleviate the negative effects (Hobfoll, 2011). Resource loss is the main component in the stress process, and COVID-19 is likely to threaten the resources of an individual from many aspects (Zhang et al., 2021). COVID-19 is similar to other stressors, in that it brings about cognitive, physical, and emotional stress (Zhang et al., 2021.).

Wuhan where the COVID-19 outbreak was first noted in China was forced to complete lockdown, which greatly changed the lifestyle of Wuhan citizens and induced a lot of pressure (Cao et al., 2020). In addition to stress over the spread of the disease, uncertainty and changes in routines also put a lot of stress on people, and studies have shown that during the COVID-19 pandemic, people are particularly vulnerable to the three main stressors: infection-related risk, activity-related risk, and financial-related risk (Park et al., 2020; Qiu et al., 2020). Based on the resource conservation theory, COVID-19 is likely to threaten the resources of an individual from many aspects, and may trigger the individual defense mechanism of self-protection, to show some irrational behavior (Hobfoll, 2011; Zhang et al., 2021). Accordingly, Individuals who perceive more epidemic stress are more likely to exhibit irrational behavior. And so is the rate of increase in cases. When there are more diagnosed cases in the area of an individual, there is a higher possibility that a person will get infected, and therefore, the strain is higher and the greater the negative effects that follow.

In addition, individual perception of disease severity, exposure factors, and individual health beliefs have a negative impact on individual compliance with preventive behavior (Zhou and Luo, 2009). The COVID-19 epidemic not only poses a serious threat to people’s physical health and lives, but also causes a variety of psychological problems, leading to an increase in various psychological distress, anxiety and depression (Benke et al., 2020; González-Sanguino et al., 2020; Qiu et al., 2020). Anxiety can also negatively affect individuals as a risk factor during the COVID-19 pandemic (Ding et al., 2021; Zhang et al., 2021). There are also studies showing that mental health has a negative impact on health behavior compliance (Grant et al., 2005; Hu, 2021). Accordingly, based on the resource protection theory, epidemy-related stressors, case growth rate, and anxiety as risk factors may threaten individuals’ resources, exhibit some irrational behaviors, and reduce compliance with epidemic prevention guidelines.

### The Impact of Protective Factors on Individuals’ Compliance With Epidemic Prevention Guidelines

In fact, not all individuals are greatly affected by stressful life or traumatic experience, and internal and external resources play an important role as a protective factor (Maschi et al., 2014). Based on the Big Five personality theory, we propose that individuals who are more conscientious and rule-abiding might be more resistant to the impact of COVID-19 and follow epidemic prevention guidelines more closely. Studies have found that the pandemic severity predicted the negative affect of people. Moreover, the relationship was moderated by conscientiousness, which means that people who are conscientious, are more resistant to the psychological impact of COVID-19 pandemic (Qiu et al., 2020). First of all, conscientiousness reflects the extent to which people are responsible and organized (Barrick and Mount, 1991), also reflects a personal characteristic resource for stress coping, they will plan and prevent themselves from maladaptive coping (Vollrath and Torgersen, 2000). Therefore, conscientious people might be more planful in coping with COVID-19, and hence, less negatively impacted. In addition, conscientious individuals are generally more immersed in work and other meaningful activities (Barrick and Mount, 1991). As a result, they may be more distant from the rate of case growth and other negative information from the media, more able to behave rationally and more compliant with epidemic prevention guidelines.

In highly stressful situations caused by the COVID-19 pandemic, protective factors from within individuals – resilient coping and hope levels – can protect individuals from less negative impacts (Ding et al., 2021). Individuals with a high level of hope have a positive attitude toward reality and the future, also actively take actions, which helps individuals better cope with negative events (Herth, 1992). Individuals with high resilience can cope with pressure in a highly adaptive way and actively solve problems (Sinclair and Wallston, 2004). When facing a series of major lifestyle changes such as forced closure management, they can cope more calmly and adapt to the new life mode more quickly. Positive psychosocial strength and proactive behaviors can help individuals cope with public health crises (Ding et al., 2021), and also have a positive impact on individuals’ compliance with preventive behavior (Zhou and Luo, 2009).

Psychological distress levels were also influenced by availability of local medical resources, efficiency of the regional public health system, and prevention and control measures taken against the epidemic situation (Qiu et al., 2020). In the early days of the COVID-19 pandemic, a shortage of medical resources and personal protective equipment is highly likely to have a negative psychological impact on individual (Bozdag and Ergun, 2020; El-Hage et al., 2020). The abundance of hospital beds and medical resources makes it easier for individuals to get medical care if they feel unwell or contract infectious diseases, so individuals are likely to have less anxiety about COVID-19 and less fear of death. Therefore, when individual resources are scarce, the number of hospital beds available, an external medical resource, may act as a protective factor to protect individuals from more rational preventive behavior during the COVID-19 pandemic. Accordingly, there are two main aims in the current study. First, we aimed to examine the impacts of internal and external risk factors (COVID-19 related perceived stress, Anxiety, Rate of case growth) on individual compliance with epidemic prevention guidelines. Second, we aimed to examine whether impact can be mitigated by the positive role of internal and external protective factors (e.g., conscientiousness, hope, resilience coping, and average number of hospital beds available).

## Materials and Methods

### Participants

The characteristics of the overall sample appear in Table 1. The sample consisted of 811 participants from Wuhan, and Jiangxia District had the greatest number of participants (*n* = 105, 12.9%), followed by Jiang’an District (*n* = 90, 11.1%) and Hongshan District (*n* = 89, 11.0%). Of the 811 participants included, 48.0% were male (*n* = 389), 52.0% were female (*n* = 422), 45.1% were 18–34 years old (*n* = 366), 32.8% were 35–54 years old (*n* = 266), 22.1% were 55 years or older (*n* = 179), 28.7% had a less than a high school degree (*n* = 233), 15.8% had a high school degree (*n* = 128), 35.6% had an associate degree (*n* = 289), 19.9% had a bachelor’s or higher degree (*n* = 161), and 55.9% (*n* = 453) were married, 44.1% (*n* = 358) were single (Table 1).

**TABLE 1:** Demographic characteristics of participants in the 13 districts (*N* = 811).

Characteristic	*n*	*%*
**District**		
Caidian	52	6.4
Qiaokou	39	4.8
Qingshan	31	3.8
Wuchang	54	6.7
Xinzhou	69	8.5
Dongxihu	76	9.4
Hannan	13	1.6
Hanyang	69	8.5
Hongshan	89	11.0
Huangpi	61	7.5
Jiang’an	90	11.1
Jianghan	63	7.8
Jiangxia	105	12.9
**Age**		
18–34	366	45.1
35–54	266	32.8
55 years or older	179	22.1
**Education**		
Less than a high school degree	233	28.7
High school degree	128	15.8
Associate degree	289	35.6
Bachelor’s degree or higher	161	19.9
**Marital status**		
Single	358	44.1
Married	453	55.9
**Gender**		
Male	389	48.0
Female	422	52.0
Other	0.00	0.00

### Procedures

A snowball sampling method was used. First, six residents from each of the 13 districts of Wuhan were selected as “first-level seeds,” and the male:female ratio was 1:1. In terms of age ranges, a ratio of 1:1:1 was used for the groups of 18–34 years old, 35∼54 years old, and 55 years old and above. Second, the “level 1 seed” forwarded the questionnaire link to their own community or community WeChat group, and their WeChat friends voluntarily and anonymously participated in the questionnaire. We received 881 surveys, of which 811 (86.2%) surveys were retained after excluding those whose IP addresses were not in Wuhan, those with an answer time longer than 15 min and those with missing data.

### Study Variables and Measures

#### Personal Level Factors

Anxiety was measured using the established, empirically validated Adult Patient-Reported Outcomes Measurement Information System (PROMIS) Short Form v1.0-Anxiety 4a (Cronbach’s alpha of 0.93) (Pilkonis et al., 2011), and the Cronbach’s alpha coefficient in this measurement was 0.951. This scale included four questions on a 5-category Likert scale that ranged from 4 to 20, with higher scores representing more anxiety.

According to a self-compiled questionnaire on COVID-19-related perceived stress from previous studies (Park et al., 2020), 27 epidemic-related stressors were measured during the COVID-19 period. This scale included twenty-seven questions on a 5-category Likert scale that ranged from 1 to 5, with higher scores indicating that individuals perceived more COVID-19-related stress. The questionnaire had good internal consistency (Cronbach’s alpha of 0.977) and convergent validity (CMIN/DF = 2.549, CFI = 0.992, NFI = 0.988, TLI = 0.979, RMSEA = 0.044).

The Mini-International Five-Factor Personality Scale was used to measure the conscientiousness level of the participants based on twenty questions on a 5-point Likert scale, and there were four questions for each of the five dimensions: extraversion, conscientiousness, agreeableness, neuroticism, and openness (Donnellan et al., 2006). Higher the score, the more obvious the trait. He Jianhua, a domestic scholar, revised the scale, and the results showed that it has good reliability and validity. Cronbach’s alpha for the questionnaire in this survey was 0.957.

Hope was measured using the 12-item Herth Hope Index, which is a valid scale with a Cronbach’s alpha of 0.97 and test-retest reliability of 0.91 (Herth, 1992). There are three dimensions: having a positive attitude toward reality and the future, taking positive actions, and maintaining close relationships with others. The total score ranges from 12 to 48, and the higher the score, the higher the level of hope. Cronbach’s alpha coefficient for the questionnaire in this survey was 0.904.

The 4-item Brief Resilient Coping Scale was used to measure the subjects’ tendency to respond to stress in a highly adaptive way (Sinclair and Wallston, 2004), using a 5-category symmetrical Likert scale with a Cronbach’s alpha of 0.76 and test-retest reliability of 0.71. This scale was able to significantly positively predict self-efficacy, positive problem solving, life satisfaction and mental health with good predictive validity. Cronbach’s alpha coefficient for the questionnaire in this survey was 0.957.

Compliance with epidemic prevention guidelines refers to the extent to which people are scientifically protected according to the recommendations in the guidelines. According to the “Guidelines for Public Protection of Novel Coronavirus Pneumonia” compiled by the Chinese Center for Disease Control and Prevention, a questionnaire for compliance with the guidelines was established to measure the protective behavior of the general population during COVID-19 prevention and control. Participants were asked to subjectively evaluate their compliance with each item during the quarantine period. According to previous study each item was scored from 0 (never done) to 100 (fully adhered to), and the total score was calculated (Park et al., 2020). The higher the score was, the better the compliance with the epidemic prevention guidelines. The questionnaire had good internal consistency (Cronbach’s alpha of 0.768) and convergent validity (CMIN/DF = 2.228, CFI = 0.984, NFI = 0.972, TLI = 0.976, RMSEA = 0.039).

#### District Level Factors

District-level factors were measured using two variables based on existing data retrieved from online resources on the daily growth rate of COVID-19 cases per 100,000 people in each district of Wuhan based on the number of confirmed COVID-19 cases from March 6, 2020, to April 26, 2020, released by the Wuhan Municipal Health Commission and the population of each district in Wuhan released by the Hubei Provincial Bureau of Statistics.

The case growth rate was used to represent the severity of COVID-19 in each district. According to the daily hospital bed usage of designated hospitals in Wuhan released by the Wuhan Municipal Health Commission from January 31, 2020, to February 25, 2020, the average number of hospital beds supplied per day in each district of Wuhan during the lockdown period was summarized.

The average number of hospital beds available represents the supply of medical resources in each district during the COVID-19 lockdown period.

#### Demographic Variables

Demographic variables included gender, age group, highest level of education and marital status.

### Statistical Analysis

We conducted descriptive analyses of demographics, district-level factors, and personal compliance with epidemic prevention guidelines. We assessed the zero-order correlations among the district-level factors and personal-level variables. We examined differences in compliance with epidemic prevention guideline variables across the districts using ANOVA tests. We analyzed the associations of district-level factors and personal-level factors with compliance with epidemic prevention guidelines using two-level, linear mixed models in a four-model testing process.

As recommended for multilevel modeling (Bryk and Raudenbush, 1992), we treated the participants (level 1) as clusters within their districts (level 2) to account for correlations among the participants from the same district. We used a random intercept test to ensure accuracy in estimating the variance. Model 1 contained only the random intercept to assess whether or not there was significant variation in compliance with epidemic prevention guidelines across districts. For Model 2, we added district-level and personal-level risk factor variables as fixed factors (rate of case growth, COVID-19-related perceived stress, anxiety). Model 3 further added district-level and personal-level protective factor variables (resilience coping, conscientiousness, hope, average number of hospital beds available). Model 4 adjusted for demographic variables (gender, age, and marital status). SPSS 25.0 and HLM 6.06 statistical software were used for the analyses.

## Results

Varied substantially across districts (*F* = 5.65, *p* < 0.001), Huangpi District experienced the greatest score at 99.525, and Qingshan District had the lowest score at 90.560 (Table 2). In addition, ANOVAs testing for differences in compliance with epidemic prevention guidelines based on gender, age, marital status and education revealed no significant differences.

**TABLE 2:** District-level variables.

District	Rate of case growth	Average number of hospital beds available	Compliance with epidemic prevention guidelines
Jianghan	0.26	426	95.97
Qiaokou	0.16	348	95.03
Wuchang	0.16	205	95.18
Hongshan	0.10	150	95.88
Qingshan	0.10	140	90.56
Hanyang	0.08	253	96.84
Jiang’an	0.06	65	97.48
Dongxihu	0.04	120	96.58
Hannan	0.04	12	97.22
Caidian	0.03	548	98.51
Jiangxia	0.03	91	98.38
Xinzhou	0.02	440	98.11
Huangpi	0.01	37	99.52

The specific results of the district-level factors, the average daily increase in confirmed COVID-19 cases per 100,000 people (case growth rate) and the average number of hospital beds available are shown in Table 2.

Correlation analysis showed that compliance with epidemic prevention guidelines was significantly correlated with COVID-19-related perceived stress, anxiety, case growth rate, hope, and conscientiousness (Table 3).

**TABLE 3:** Means, standard deviations, and correlation between personal-level and district-level variables.

	M ± SD	1	2	3	4	5	6	7	8
1. Compliance with epidemic prevention guidelines	96.96.42	1							
2. COVID-19-related perceived stress	2.370.87	−0.16**	1						
3. Anxiety	1.850.92	−0.18**	0.47**	1					
4. Case growth rate	0.080.07	−0.16**	–0.03	0.01	1				
5. Resilience coping	3.860.89	0.06	−0.13**	−0.21**	−0.11**	1			
6. Hope	3.190.50	0.14**	−0.16**	−0.29**	−0.09**	0.71**	1		
7. Conscientiousness	13.162.00	0.12**	−0.08*	−0.21**	0.02	0.29**	0.32**	1	
8. Average number of hospital beds available	178.91147.70	–0.05	0.01	–0.03	0.55**	−0.08*	–0.03	–0.01	1

*p < 0.05; **p < 0.01.

The results of the multilevel linear mixed model analysis are depicted in Table 4. Model 1, with no independent variables, showed statistically significant results, which suggests that compliance with epidemic prevention guidelines differs based on the characteristics of the individual’s district (ICC = 0.091, *P* < 0.001). The intraclass correlation indicated that 9.1% of the variance in compliance with epidemic prevention guideline scores was due to differences across districts. It is necessary to take into consideration both individual-level and district-level variables and how they affect each other related to compliance with epidemic prevention guidelines. In model 2, risk factor variables at the personal level (COVID-19-related perceived stress, anxiety) and district level (rate of case growth) were added to model 1. In the analysis, all the risk factor variables had a significant effect on compliance with epidemic prevention guidelines. In model 2, living in a district with a higher rate of case growth was associated with a significant reduction in compliance with epidemic prevention guidelines (β = −16.084, *p* = 0.023), as did the presence of higher levels of COVID-19-related perceived stress (β = −0.844, *p* = 0.002) and anxiety (β = −0.725, *p* = 0.008).

**TABLE 4:** Regressions of district-level and personal factors on compliance with epidemic prevention guidelines using multilevel modeling.

	Model 1	Model 2	Model 3	Model 4
Intercept	96.617 (0.570)***	101.405 (0.599)***	94.678 (2.469)***	94.392 (2.394)***
**Risk factors**				
*District level*				
Rate of case growth		-16.084 (6.070)*	−19.204 (5.707)**	−19.008 (5.778)**
**Personal level**				
COVID-19 related perceived stress		−0.844 (0.270)**	−0.868 (0.261)***	−0.834 (0.242)***
Anxiety		−0.725 (0.272)**	−0.442 (0.250)	−0.444 (0.264)
**Protective factors**				
*Personal level*				
Resilience coping			−0.774 (0.472)	−0.793 (0.454)
Conscientiousness			0.274 (0.129)*	0.306 (0.140)*
Hope			1.844 (0.730)**	2.027 (0.739)**
*District level*				
Average number of hospital beds available			0.003 (0.001)	0.003 (0.002)
**Demographic variables**				
*Gender*				
Male				−0.207 (0.488)
Female				Reference
*Marital status*				
Single				3.669 (5.146)
Married				Reference
*Age*				
18–34				−4.848 (5.165)
35–55				−0.259 (0.326)
55 years or older				Reference
**Random variance components**				
Between-group intercept	3.850***	1.915***	2.357***	2.394***
Within group	38.549	37.388	36.711	36.465

*p < 0.05; **p < 0.01; ***p < 0.001.

In model 3, the protective factor variables were added to model 2. We found that participants with a higher conscientiousness score had significantly greater compliance with epidemic prevention guidelines (β = 0.274, *p* = 0.033), as did participants with a higher hope scores (β = 1.844, *p* = 0.012). Notably, after adding protective factors into the model, the rate of case growth (β = −19.204, *p* = 0.008) and COVID-19-related perceived stress (β = −0.868, *p* = 0.001) still had a significant negative effect on compliance with epidemic prevention guidelines, but the negative predictive effect of anxiety factors disappeared. The associations between the average number of hospital beds available and resilience coping were not significant. Finally, the demographic factor variables were added in model 4, and the results were the same as those in model 3.

## Discussion

The current study aims to compare compliance with epidemic prevention guidelines among citizens from 13 districts in Wuhan and to explore the influence of individual psychological factors and district-level factors on compliance. An analysis of the characteristics of public health emergencies in Hubei Province shows that the COVID-19 outbreak is a major public health event, and negative factors such as severity, uncertainty and other negative factors cause anxiety, depression and other negative psychological manifestations in individuals. This investigation revealed significant differences in scores related to compliance with the epidemic prevention guidelines among all districts of Wuhan. The scores of citizens in Qingshan District were the lowest, and those in Huangpi District were the highest. Findings of the current study revealed that the risk factors case growth rate, COVID-19-related perceived stress, anxiety, significantly negatively predicted compliance. After protective factors (resilience coping, conscientiousness, hope and average number of hospital beds available) were added, hope and conscientiousness significantly positively predicted compliance with epidemic prevention guidelines, and the negative predictive effect of anxiety disappeared. After the addition of control variables, this result remained the same.

### Impact of Risk Factors on Individuals’ Compliance With Epidemic Prevention Guidelines

In this study, individuals’ COVID-19-related perceived stress and anxiety levels and the growth rate of cases at the district level had a significant negative predictive effect on compliance with epidemic prevention guidelines. When individuals perceive more stressors and anxiety related to the epidemic and an increased rate of cases in their district, their compliance with epidemic prevention guidelines is worse. According to resource conservation theory, either potential or actual resource loss can trigger adverse reactions in individuals (Hobfoll, 2011). When people face such a desperate situation of resource depletion, this may also trigger their self-protection defense mechanism, thus leading them to display some irrational behaviors. Many internal and external risk factors (COVID-19-related perceived stress, anxiety, case growth rate) during the COVID-19 pandemic lockdown may cause individuals to be in a state of resource loss and may affect whether universal values or specific resources are affected to a certain degree. When this loss state reaches a certain degree, the probability of rational coping decreases for individuals, so they perform poorly in terms of goal-oriented protective behavior.

### Impact of Protective Factors on Individuals’ Compliance With Epidemic Prevention Guidelines

This study also found that conscientiousness and hope have a significant positive effect on compliance with epidemic prevention guidelines, and the negative effect of anxiety on compliance with epidemic prevention guidelines disappeared when protective factors were added. Resource conservation theory assumes that resources exist within and outside of the individual, namely, at different levels, and determines how effectively individuals cope with stress in negative events (Hobfoll, 2011). People use key resources to cope with stressful situations in the current environment to alleviate negative impacts. Individuals have many protective resources, such as stable personality traits and positive psychosocial strengths. These protective factors can protect individuals from excessive arousal, promote recovery from stress and lead to better performance in subsequent tasks (Wolfgang and Paul, 2011). In addition, in the AB5C model of personality, conscientiousness has nine dimensions, including strict rationality and orderliness, and individuals with conscientiousness are more able to comply with social requirements and norms to control their behaviors (Hofstee et al., 1992; John and Srivastava, 1999). Therefore, during the COVID-19 pandemic lockdown, people with conscientiousness are more able to follow the epidemic prevention guidelines and behave better.

Finally, this study originally assumed that the average number of hospital beds available as a protective social resource could alleviate the negative impact, but the impact was not significant in this survey. The reason may be that the average number of hospital beds available in each district reflects not only the social resources of the district but also the serious situation of the epidemic to a certain extent, which may weaken the level of representability relative to the average number of hospital beds available and the degree to which social resources are a factor.

## Implication

The research results have the following implications: First, when people face a major public health event, negative information at the district level may affect their ability to respond positively. At this time, the output of protective resources at the government level should be increased to provide more social support for the public, which may help to alleviate the negative impact of anxiety and other negative factors. Second, in the face of major stressful events, an individual’s sense of conscientiousness and hope play an important and positive role. We should pay attention to cultivating our own strong self-control and strict self-discipline to comply with social requirements and build hope such as community-wide mindfulness-based stress reduction training and popularize positive psychology for the public.

## Limitations

This study also has many limitations, which should be addressed in subsequent studies. First, the representativeness of protective factor index selection at the district level needs to be considered. Future research is needed to expand data collection channels and further explore the influence of protective factors at the district level. Second, the negative effect of anxiety disappeared after the addition of protective factors, which suggests that the specific mechanism of action between variables can be further explored in subsequent studies. Finally, a cross-sectional study could collect dynamic data on compliance with epidemic prevention guidelines and psychological changes in multiple time periods in the future, explore whether protective factors within individuals play a positive role in each stage of event development, and determine the causal relationships among variables.

## Conclusion

Overall, findings of the current study suggest significant variations in people’s compliance with epidemic prevention guidelines across districts. Internal and external risk factors (COVID-19 related perceived stress, Anxiety, Rate of case growth) have a negative impact on individual compliance with epidemic prevention guidelines, and this impact can be mitigated by the positive role of internal and external protective factors(conscientiousness, Hope). This may assist in providing empirical evidence for related theories of individual compliance with health behavior under stressors, also providing a reference to effectively buffer against anxiety for individuals facing under stressful events and promote compliance with epidemic prevention guidelines.

## Data Availability Statement

The raw data supporting the conclusions of this article will be made available by the authors, without undue reservation.

## Ethics Statement

The studies involving human participants were reviewed and approved by the Ethics Committee of Wuhan Sports University (2020004). The patients/participants provided their written informed consent to participate in this study.

## Author Contributions

XW: conception and design of study and revising the manuscript for important intellectual content. YC: conception and design of study, acquisition and analysis of the data, and drafting the manuscript. Both authors contributed to the article and approved the submitted version.

## Conflict of Interest

The authors declare that the research was conducted in the absence of any commercial or financial relationships that could be construed as a potential conflict of interest.

## Publisher’s Note

All claims expressed in this article are solely those of the authors and do not necessarily represent those of their affiliated organizations, or those of the publisher, the editors and the reviewers. Any product that may be evaluated in this article, or claim that may be made by its manufacturer, is not guaranteed or endorsed by the publisher.
